# Supplementary material for: Enhanced Risk Stratification in Early-Stage Endometrial Cancer: Integrating POLE through Droplet Digital PCR and L1CAM
Source: Cancers (Basel). 2023 Oct 9;15(19):4899. doi: 10.3390/cancers15194899 (PMC10571976; doi:10.3390/cancers15194899)
Supplement: Supplementary file 1 [file cancers-15-04899-s001.zip › Table S1-final revision.pdf]

Table S1. Clinicopathological characteristics according to molecular classification in early-stage endometrial cancer patients (n=183).

Characteristics	n=183	*POLEmut* n=29	MMR-D n=53	p53abn n=16	NSMP n=76	*p*-value
Age						0.098
< 60	134	22 (16.4%)	43 (32.1%)	8 (6.0%)	61 (45.5%)	
≥ 60	49	7 (14.3%)	10 (20.4%)	8 (16.3%)	24 (49.0%)	
OP						0.520
Hysterectomy	7	0 (0.0%)	2 (28.6%)	1 (14.3%)	4 (57.1%)	
Hys+BSO	19	1 (5.3%)	4 (21.1%)	2 (10.5%)	12 (63.2%)	
Hys+BSO+LD	157	28 (17.8%)	47 (29.9%)	13 (8.3%)	69 (43.9%)	
Histologic type						<0.001
Endometrioid	166	29 (17.5%)	51 (30.7%)	4 (2.4%)	82 (49.4%)	
Non-endometrioid	17	0 (0.0%)	2 (11.8%)	12 (70.6%)	3 (17.6%)	
Histologic grade						<0.001
Low	146	24 (16.4%)	43 (29.5%)	4 (2.7%)	75 (51.4%)	
High	37	5 (13.5%)	10 (27.0%)	12 (32.4%)	10 (27.0%)	
LVSI						0.223
Absent	152	26 (17.1%)	40 (26.3%)	15 (9.9%)	71 (46.7%)	
Present	31	3 (9.7%)	13 (41.9%)	1 (3.2%)	14 (45.2%)	
Myometrial invasion						0.324
<50%	145	24 (16.6%)	40 (27.6%)	14 (9.7%)	67 (46.2%)	
>50%	38	5 (13.2%)	13 (34.2%)	2 (5.3%)	18 (47.4%)	
FIGO stage 2009						0.718
IA	133	23 (17.3%)	36 (27.1%)	14 (10.5%)	60 (45.1%)	
IB	33	5 (15.2%)	11 (33.3%)	0 (0.0%)	17 (51.5%)	
II	15	1 (6.7%)	5 (33.3%)	2 (13.3%)	7 (46.7%)	
III	2	0 (0.0%)	1 (50.0%)	0 (0.0%)	1 (50.0%)	
FIGO stage updated 2023						<0.001
IA	109	20 (18.3%)	29 (26.6%)	4 (3.7%)	56 (51.4%)	
IB	18	2 (11.1%)	6 (33.3%)	0 (0.0%)	10 (55.6%)	
IC	8	0 (0.0%)	2 (25.0%)	2 (25.0%)	4 (50.0%)	
IIA	9	1 (11.1%)	2 (22.2%)	0 (0.0%)	6 (66.7%)	
IIB	10	1 (10.0%)	6 (60.0%)	0 (0.0%)	3 (30.0%)	
IIC	29	5 (17.2%)	8 (27.6%)	10 (34.5%)	6 (20.7%)	
Prognostic risk group*						<0.001
Low	100	20 (20.0%)	28 (28.0%)	3 (3.0%)	49 (49.0%)	
Intermediate	20	3 (15.0%)	6 (30.0%)	2 (10.0%)	9 (45.0%)	
High intermediate	47	6 (12.8%)	17 (36.2%)	1 (2.1%)	23 (48.9%)	
High	16	0 (0.0%)	2 (12.5%)	10 (62.5%)	4 (25.0%)	
Adjuvant treatment						0.058
None	147	25 (17.0%)	41 (27.9%)	12 (8.2%)	69 (46.9%)	
Radiotherapy	25	4 (16.0%)	8 (32.0%)	0 (0.0%)	13 (28.6%)	
Chemotherapy	7	0 (0.0%)	2 (28.6%)	3 (42.9%)	2 (52.0%)	
Chemoradiotherapy	4	0 (0.0%)	2 (50.0%)	1 (25.0%)	1 (25.0%)	

BSO, Bilateral salpingo-oophorectomy; LD, Lymph node dissection; FIGO, International Federation of Gynecology and Obstetrics; LVSI, lymphovascular space invasion; MMR-D, mismatch repair-deficient; p53abn, p53 mutated; NSMP, no specific molecular profile; *POLEmut*, DNA polymerase epsilon-mutated;*risk stratified according to the European Society for Medical Oncology (ESMO), European Society for Radiotherapy & Oncology (ESTRO) and European Society of Gynaecological Oncology (ESGO) consensus guideline.
