# Supplementary figures and images for: Enhanced Risk Stratification in Early-Stage Endometrial Cancer: Integrating POLE through Droplet Digital PCR and L1CAM
Source: Cancers (Basel). 2023 Oct 9;15(19):4899. doi: 10.3390/cancers15194899 (PMC10571976; doi:10.3390/cancers15194899)

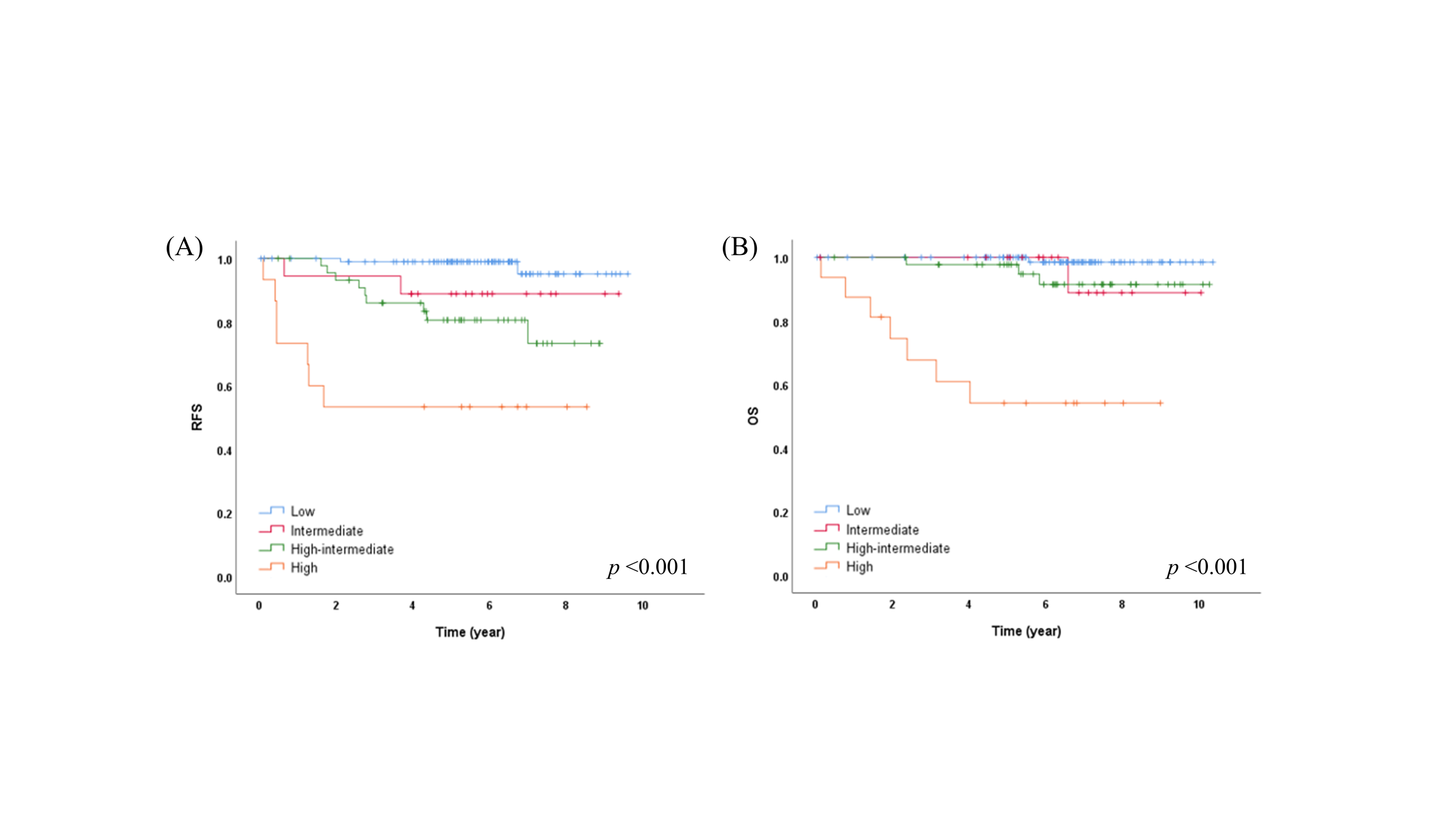

Supplement: Supplementary file 1 [file cancers-15-04899-s001.zip › Figure S1.TIF]
